# 

**Published:** 1919-07-19

**Authors:** 


### Metropolitan Hospital Sunday Fund.

Up to July 11 £56,000 had been received at the Mansion House. This sum includes: The Rt. Hon. the Lady Strathcona and Mount Royal, £3,000; St. Mark's, North Audley Street, £502; St. Columba's, Church of Scotland, Pont Street, £575; St. James's, Piccadilly, £380; Christ Church, Lancaster Gate, £296; Holy Trinity, Sloane Street, £278; Brompton Parish Church, £230; St. Paul's, Knightsbridge, £227; St. Peter's, Eaton Square, £214; Brixton Independent Church, £206; St. Simeon's, Upper Chelsea, £173; St. Alphage, London Wall, with St. Mary, Aldermanbury, £166; St. George's, Hanover Square, £157; Wimbledon Churches, £154; St. Jude's, Kensington, £146; Westminster Chapel, £141; St. Stephen's, Avenue Road, £132; St. Stephen's, Gloucester Road, £130; Holy Trinity, Kennington Gate, £122; Greek Church, Bayswater, £121; Essex Church, Kensington, £119; All Saints', Ennismore Gardens, £116; St. Peter's, Cranley Gardens, £109; Christ Church, Mayfair, £108; Cannon Brewery Co., Ltd., £105; St. Mary-in-the-Boltons, £103; Temple Church, £102.

### Gifts and Bequests.

MISS CATHERINE NEILD, of Oldham, left £500 to Oldham Infirmary.

MR. STEPHEN CHIVERS, of Cambridge, bequeathed £1,000 to Addenbrooke's Hospital.

THREE HUNDRED POUNDS was bequeathed to the Johnstone District Cottage Hospital by Mr. A. H. Renshaw, of London and Oxford.

MR. WILLIAM HIPKINS, of Leamington Spa, left £300 to the Warneford Hospital, Leamington, and to the Hospital for Women, Wolverhampton.

MR. THOMAS JOSEPH WILLIAMS, of Bristol, left £2,000 to the Bristol Infirmary, £1,000 to the Bristol General Hospital, and £300 to the Bristol Eye Hospital.

MR. FRANK ROBINSON, of Hurstpierpoint, Sussex, left £1,000 each to the Crawley and Ifield Hospital, and the Miller Hospital, Greenwich, and £500 to the Redhill and Reigate Hospital.

MRS. MCCOSH, of Cairnhill House, Coatbridge, has intimated her intention of making a gift of £10,000 for the provision of a children's ward in the Coatbridge Hospital as a memorial to her husband.

MR. EDWARD LYTTLETON GETHING, of Bournemouth, among numerous charitable bequests left £2,000 each to Cardiff Infirmary, Newport (Monmouthshire) Hospital, Asylum for the Blind, Bristol, and the London Lock Hospital.

MR. W. BRINDLEY, of Bournemouth, has left £50 to the South London Ophthalmic Hospital.

MR. and MRS. J. J. RICHARDSON, of Selby, have given £2,000 to Selby War Memorial Hospital.

THE Chelsea Hospital for Women has received a donation of £200 from the Lady Strathcona.

UNDER the will of the late Captain T. A. M. Dickin, a legacy of £300 has been left to the Salop Royal Infirmary and one of £100 to the Eye, Ear, and Throat Hospital, Shrewsbury.

To a hospital or institution for blind or crippled soldiers in the war, to be chosen by his executor, Mr. Hugh Bagot has bequeathed £1,000.

ELEANORA LADY TREVELYAN has left £1,000 each to the British Red Cross Society and the London Hospital, and £100 to St. George's Hospital.

MRS. A. W. CLEGG has given £1,000 to the Rochdale Infirmary to endow a bed in remembrance of her mother's happy association with the institution.

MRS. ELIZABETH SARAH ASTON, of Handsworth, left £1,000 to the Birmingham General Dispensary, and the same amount to the Birmingham Children's Hospital. Subject to other legacies, the residue of her estate, the value of which is £23,022, goes to the Birmingham Children's Hospital.

